# Nanotechnology in reproductive medicine: from gamete engineering to precision therapeutics

**DOI:** 10.1039/d6ra01614f

**Published:** 2026-07-08

**Authors:** Xu Wen, Zhiyan Wang, Jiahui Lin, Longjie Li, Haiyun Wang, Pei Liu, Hao Hu, Chao He, Zijia Zheng, Ruisi Liu, Kejun Dong, Donghui Huang, Xianjin Xiao

**Affiliations:** a Institute of Reproductive Health, Tongji Medical College, Huazhong University of Science and Technology Wuhan 430030 China xiaoxianjin@hust.edu.cn jhsyyjs@126.com; b The Second Clinical School, Tongji Hospital, Tongji Medical College, Huazhong University of Science and Technology Wuhan 430030 China; c Reproductive Medical Center, Zhongnan Hospital of Wuhan University Wuhan 430071 China; d Hubei Provincial Key Laboratory of Developmentally Originated Diseases Wuhan 430071 China; e School of Life Science and Technology, Wuhan Polytechnic University 430023 Wuhan China; f Department of Obstetrics and Gynecology, Union Hospital, Tongji Medical College, Huazhong University of Science and Technology Wuhan 430022 China 540151069@qq.com

## Abstract

Infertility represents a significant global health burden, necessitating advanced therapeutic interventions. While Assisted Reproductive Technologies have revolutionized fertility treatment, they remain constrained by limited efficacy, off-target toxicity, and procedural complexity. Nanotechnology has emerged as a transformative frontier, offering precise targeting and controlled release capabilities to circumvent these limitations. This review systematically synthesizes nanotechnological applications in reproductive medicine, ranging from gamete quality enhancement—*via* antioxidant nanoparticles, microfluidic sperm selection and improved cryopreservation—to targeted therapies for complex disorders including premature ovarian insufficiency, polycystic ovary syndrome, and endometriosis. Despite promising preclinical outcomes, clinical translation is impeded by challenges regarding biocompatibility, large-scale manufacturing, and ethical regulation. Future perspectives emphasize the integration of multi-omics, artificial intelligence, and biocompatible material innovation to elucidate molecular mechanisms and accelerate the transition from bench to bedside, ultimately advancing precision reproductive medicine.

## Abstract 2

This review summarizes nanotechnological applications in reproductive medicine, spanning sperm selection, oocyte cryopreservation, and targeted theranostics for gynecological disorders like endometriosis and ovarian diseases.

## Graphical abstract

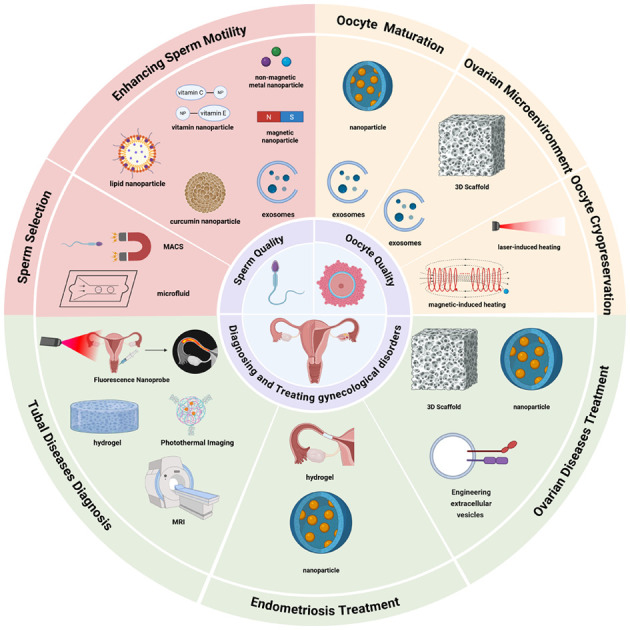

## 1 Introduction

Reproductive dysfunction and consequent infertility in both sexes have emerged as escalating global public health challenges, profoundly impacting individual reproductive goals, familial well-being, and population stabilitystitutes the cornerstone of reproductive health. Specifically, sperm parameters (motility, morphology, and DNA integrity) and oocyte developmental competence are critical determinants of successful fertilization, embryogenesis, and clinical pregnancy outcomes.^1^ However, gamete integrity and function are vulnerable to a myriad of intrinsic and extrinsic insults. Factors such as oxidative stress, genetic aberrations, environmental pollutants, age-related decline, and adverse lifestyle habits can compromise these cells, thereby serving as primary drivers of fertility disorders.^2^

Infertility stemming from compromised gamete quality remains a persistent clinical challenge. While assisted reproductive technologies (ART)—notably *in vitro* fertilization (IVF) and intracytoplasmic sperm injection (ICSI)—have revolutionized the management of infertility, current therapeutic frameworks are constrained by inherent limitations. Standard protocols are not only intricate and cost-prohibitive but are also contingent upon baseline sperm parameters. Consequently, clinical efficacy is often compromised in cases of severe asthenozoospermia, teratozoospermia, or idiopathic etiologies. Furthermore, critical bottlenecks remain regarding the efficiency of *in vitro* maturation (IVM), the preservation of gamete viability during cryopreservation, and the mitigation of inflammation within the reproductive tract.^3^

Against this backdrop, nanotechnology has garnered significant attention owing to its unique physicochemical versatility. Key attributes—including ultra-high specific surface area, tunable surface chemistry, biocompatibility, and superior drug-loading capacity—render nanomaterials particularly suitable for reproductive applications.^4^ Emerging research highlights the therapeutic potential of these materials in preserving gamete competence. For instance, antioxidant nanoparticles effectively scavenge excessive ROS, thereby mitigating oxidative damage to gametes.^5^ Furthermore, delivery systems utilizing liposomes, polymeric nanoparticles, or exosomes enable the precise transport of bioactive payloads, such as hormones, growth factors, and gene regulatory elements. These platforms have been shown to enhance oocyte maturation, optimize cryoprotection protocols, and support early embryogenesis.^6^ Beyond these applications, nanotechnology is driving advances in high-throughput sperm sorting, the modulation of the ovarian microenvironment, and targeted pharmacotherapy within the reproductive tract.

Several previous reviews have discussed the application of nanotechnology in reproductive medicine, medically assisted reproduction, infertility treatment, and female reproductive healthcare. Earlier work introduced the emerging use of nanomaterials in reproductive biology and clinical reproductive medicine, while subsequent reviews emphasized ART-related applications, clinical translation opportunities, and the benefits and challenges of nanomaterials in assisted reproduction.^7–11^ More recent reviews have further summarized nanoparticles in women's reproductive health and broader reproductive healthcare, including applications in PCOS, endometriosis, uterine disorders, sexually transmitted infections, imaging, and drug delivery.^12,13^ Nevertheless, many existing reviews focus on either general reproductive nanomedicine, selected ART procedures, or specific female reproductive diseases. A materials-centered and disease-integrated synthesis that links nanoplatform design, gamete quality control, reproductive microenvironment regulation, safety considerations, and translational barriers across both male and female infertility remains relatively limited.

In this review, we systematically summarize recent advances in nanotechnology for optimizing gamete quality and improving the diagnosis and management of reproductive disorders. Compared with previous reviews, the present article emphasizes the continuum from gamete engineering to precision therapeutics, covering sperm quality improvement, sperm selection, oocyte maturation, cryopreservation, ovarian dysfunction, polycystic ovary syndrome, tubal disease, and endometriosis. Particular attention is given to the chemistry and materials science aspects of representative nanoplatforms, including material composition, particle size, surface functionalization, loading capacity, release behavior, biodegradability, and structure–activity relationships. We further evaluate their mechanisms of action, therapeutic efficacy, limitations, reproductive safety concerns, scalability, and translational readiness. By integrating material design, reproductive biology, preclinical evidence, and clinical barriers, this review aims to provide a balanced framework for the rational development and cautious clinical translation of nanotechnology-enabled reproductive medicine. To facilitate cross-platform comparison, a comprehensive summary of representative nanoplatforms is provided in Table S1 (SI).

## 2 Research advances in nanotechnology for sperm quality improvement and optimization

Male infertility constitutes a significant public health concern, impacting the well-being of many families. Research indicates that oxidative stress may impair sperm motility and function, thereby contributing to male infertility.^14^ Consequently, enhancing sperm quality represents a critical approach to addressing male infertility. Although traditional ART methods such as IVF have achieved considerable success in treating specific infertility issues, their efficacy remains limited for idiopathic infertility and demands high-quality sperm.^15^ In contrast, nanotechnology offers distinct benefits. Nanomaterials including cerium dioxide nanoparticles (CeO_2_NPs), selenium nanoparticles (SeNPs), and zinc oxide nanoparticles (ZnONPs) work by neutralizing free radicals and reducing inflammation. They enable precise delivery of bioactive molecules, thereby promoting sperm maturation.^16^ These capabilities offer infertility patients more minimally invasive and accurate treatment options, and when combined with ART, hold potential to significantly enhance fertility outcomes.

### 2.1 Exploration of nanomaterials in enhancing sperm motility

Male infertility arises from diverse causes, with oxidative stress in sperm representing a significant factor. ROS such as superoxide anions and hydroxyl radicals are major contributors to male infertility.^17^ When intracellular ROS exceed antioxidant defense capacities, oxidative stress ensues, damaging sperm membranes and DNA, reducing motility, and impairing morphology and function.^18^ This further affects embryonic development and increases miscarriage risk.^19^ Currently, various nanomaterials, such as CeO_2_NPs, SeNPs, and ZnONPs, have demonstrated the potential to enhance sperm motility and cytoprotective functions, thereby offering novel therapeutic avenues for the treatment of male infertility through antioxidant and anti-inflammatory.

#### 2.1.1 Nanoparticles

**Fig. 1:**
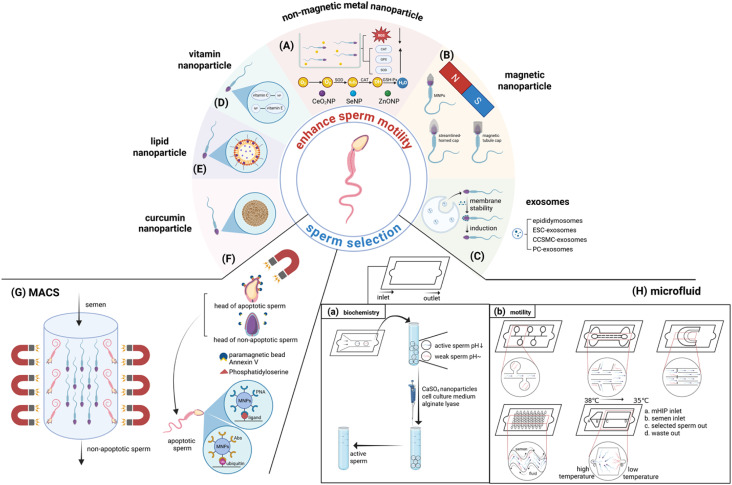
Explorations of nanomaterials in enhancing sperm motility. (A) Non-magnetic metallic nanoparticles. (B) Magnetic nanoparticles. (C) Exosomes. (D) Nanovitamins. (E) Liposomal nanoparticles. (F) Curcumin nanoparticles. (G) Magnetoactivated cell sorting. (H) Microfluidics. (a) Biochemical methods. (b) Motility assessment.

CeO_2_NPs exhibit antioxidant activity, mimicking the function of antioxidant enzymes by enhancing the activity of catalase and superoxide dismutase.^20^ Concurrently, CeO_2_NPs effectively counteract ROS, significantly reducing levels of lipid peroxidation products and thereby mitigating oxidative stress in sperm.^21^ Falchi *et al.* demonstrated that low-dose CeO_2_NPs enhance sperm survival and function by oxygen storage and ROS scavenging.^22^ Similarly, SeNPs improve sperm motility *via* antioxidant mechanisms, particularly in cryopreserved sperm applications, where their antioxidant properties eliminate ROS within spermatozoa and protect them from oxidative damage.^23^ Khalil *et al.* observed that SeNPs enhanced sperm motility, mitochondrial function, and membrane integrity in bulls and sheep. Concurrently, SeNPs boosted glutathione peroxidase and catalase activity, thereby further elevating cellular antioxidant capacity to sustain normal metabolism. These alterations collectively promote sperm development.^24^ High-temperature environments induce ROS production in the testicular microenvironment, exacerbating male infertility. El-Gindy *et al.* found that ZnONPs supplementation mitigates high-temperature effects on sperm quality, enhancing antioxidant capacity and sperm motility.^25^ Khalil *et al.* further demonstrated that ZnONPs also enhance sperm motility and mitochondrial activity, improve sperm motility, counteract oxidative stress, and boost fertilization capacity.^26^ These non-magnetic metal nanoparticles play a pivotal role in strengthening overall sperm quality and fertilization capacity, showcasing the application potential of nanomaterials in sperm protection (Fig. 1A).

Magnetic nanoparticles, typically iron oxides, allow remote control through binding to the sperm head, thereby enhancing sperm quality.^27^ Owing to their excellent biocompatibility, these magnetic nanoparticles are frequently employed in hybrid systems for sperm micromotors to promote sperm motility or deliver drugs.^28^ The magnetic nanoparticle-based sperm micromotor designed by Striggow *et al.* utilizes static magnetic fields to guide sperm towards directed movement.^29^ The motor developed by Xu *et al.* employs powerful propulsion to induce sperm to swim against blood flow, with precise control of sperm movement achieved *via* external magnetic fields.^30^ These magnetic nanoparticles offer novel avenues for enhancing sperm function and treating male infertility (Fig. 1B).

Whilst the antioxidant properties of nanoparticles may enhance sperm motility, they may adversely affect male germ cells and other somatic cells. For instance, prolonged exposure to cerium oxide nanoparticles CeO_2_NPs has been shown to impair male reproductive function, resulting in decreased sperm count and motility.^31^ Silver nanoparticles can cause reduced sperm count and motility in male rats.^32^ Długosz *et al.* proposed that modifying particle size and shape, along with surface modifications, could reduce or control the toxicity of metallic nanoparticles and metal oxides.^33^ Consequently, future research should prioritize enhancing nanoparticle biocompatibility, as well as optimizing dosage and delivery strategies, to maximize antioxidant benefits while minimizing potential cytotoxic risks.

#### 2.1.2 Exosomes

Exosomes are secreted cellular structures composed of phospholipid bilayers, typically measuring 40 to 150 nm in diameter. They play a crucial role in intercellular communication by conveying bioactive proteins, lipids, and RNA,^34^ and exhibit therapeutic efficacy in enhancing sperm motility.^35^ The epididymal corpus luteum facilitates the transport of proteins and MicroRNAs (miRNAs) essential for fertilization through rapid adhesion and fusion with spermatozoa.^36^ Yue *et al.* demonstrated that human umbilical cord mesenchymal stem cell-derived exosomes (hUCMSC-exosomes) improve sperm motility, reduce ROS levels, decrease apoptosis-associated protein expression, and significantly enhance sperm vitality.^37^ Exosomes also address cell membrane damage induced by sperm cryopreservation.^38^ Hezavehei *et al.* engineered glycerophospholipid (GPL) micelles and cholesterol-loaded cyclodextrin (CLC) micelles, averaging 138 nm and 124 nm in size respectively, which markedly enhanced the function and survival rate of cryopreserved sperm.^39^ Compared to potentially toxic nanoparticles, stem cell-derived exosomes offer high safety and precise delivery, aiding sperm survival in complex reproductive environments.^40^ Nevertheless, scaling up exosome production and refining purification techniques remain essential for ensuring successful clinical translation of exosome therapies. Concurrently, enhancing exosome stability *in vivo* constitutes an urgent challenge, with these factors collectively limiting their widespread application (Fig. 1C).

#### 2.1.3 Nanoparticle vitamins

Both vitamin E and vitamin C nanoparticles exhibit potent antioxidant capabilities, reducing sperm damage while enhancing motility and survival rates. Sánchez *et al.* observed that vitamin E incorporated into nanoemulsions significantly reduced ROS levels, with high-concentration treatments markedly improving sperm motility and survival.^41^ Nano-vitamin C also markedly increases sperm count and enhances motility.^42^ Although nano-vitamins are widely applied in animal-assisted reproductive technologies, human infertility treatment cases remain scarce. Limited research exists on their safety and efficacy, with insufficient clinical trial support necessitating further exploration and validation (Fig. 1D).

### 2.2 Strategies for sperm function restoration *via* nanotechnology

Male infertility arises from complex etiologies, primarily categorized as congenital, acquired, or idiopathic. While existing ART offers fertility solutions for many infertile couples, limitations persist.^43^ Approximately 15% of couples are diagnosed with idiopathic infertility, which ART struggles to address.^44^ For severe male factor infertility or untreated bilateral tubal obstruction, IVF becomes the sole option, limiting ART applicability. Concurrently, patients with high sperm DNA fragmentation rates frequently experience ICSI treatment failure, necessitating testicular sperm extraction to enhance success rates. Furthermore, embryo implantation and pregnancy rates remain low in cases of severe oligoasthenozoospermia or azoospermia.^45^

In recent years, liposomal nanocarrier delivery technology has offered novel strategies for improving sperm quality and treating male infertility. Nanocarriers enable precise delivery of drugs, proteins, mRNA, or extracellular vesicles (EVs). By binding specifically to germ cells, they promote sperm maturation and functional recovery, opening new avenues for male infertility treatment.^46^

#### 2.2.1 Nanotherapy for congenital factors

Infertility caused by congenital factors mostly involves genetic defects and developmental problems, like Y chromosome microdeletions and genetic mutations. These factors cause male infertility, where conventional ART treatments yield limited efficacy.^47^ Nanocarriers, leveraging their microscopic dimensions and precise delivery capabilities, offer novel approaches in this field. Dmc1 deficiency leads to azoospermia; mRNA therapy can correct the genetic defect, but delivery efficiency remains low.^48^ CAP lipid nanoparticles (LNPs) developed by Du *et al.* enhanced delivery efficiency,^49^ successfully restoring spermatogenesis in mouse models. Pin1 deficiency impairs testicular development and spermatogenesis; Kim *et al.* employed cellulose nanoparticle-encapsulated cationic lipid complexes for direct Pin1 protein delivery, partially restoring sperm function in Pin1-negative male mice.^50^ Whilst liposomal nanocarriers can ameliorate sperm dysfunction caused by genetic defects, research remains limited for hereditary infertility stemming from chromosomal abnormalities or androgen insensitivity. Future studies should focus on expanding the target range of nanodelivery systems, enhancing their gene editing and regulatory capabilities, whilst deepening the understanding of spermatogenesis molecular mechanisms. This will provide novel therapeutic strategies for male infertility arising from diverse genetic factors (Fig. 1E).

#### 2.2.2 Nanotherapy for acquired factors

Varicocele is a common cause of male infertility, leading to testicular hyperthermia and oxidative stress that impair sperm quality.^51^ It affects 15–40% of infertile men globally, with surgical treatment being complex and carrying risks.^52^ Nanotechnology offers minimally invasive therapeutic options. Exosomes, as nanovesicles, can fuse with sperm membranes to release antioxidant enzymes and restore sperm motility.^53^ Research by Sadraei *et al.* demonstrated functional defects in exosomes from varicocele patients. Treatment of varicocele rats with exosome-encapsulated nano-curcumin significantly improved sperm concentration and motility while markedly reducing abnormal morphology rates.^54^ Exosome-encapsulated nanoparticle technology shows promising efficacy in treating acquired infertility, though challenges remain in exosome extraction and application (Fig. 1F).

### 2.3 Nanoscale solutions for sperm selection and optimization

The success of assisted reproductive technology (ART) depends heavily on the effectiveness of sperm selection strategies. While intracytoplasmic sperm injection (ICSI) permits fertilization even with suboptimal sperm quality,^55^ identifying and isolating the most functional spermatozoa remains essential for maximizing clinical outcomes.^56^

Standard sperm selection techniques employed in ART laboratories include the swim-up method (SU) and density gradient centrifugation (DGC).^57^ These straightforward and cost-effective techniques enhance the efficiency of selecting motile, morphologically normal spermatozoa while separating sperm from other cells and toxic substances, thereby reducing ROS production. However, conventional methods have limitations: although SU is simple to perform, it yields low sperm recovery rates, exhibits poor efficiency, and generates ROS that may impair sperm quality; DGC exhibits reduced efficiency under high viscosity conditions and generates increased ROS, potentially causing aggregation of sperm with other cells and compromising separation efficacy.^58^ These issues significantly impact assisted reproductive success rates.

In recent years, nanotechnology has been progressively applied to sperm selection. This enables differentiation between non-apoptotic and apoptotic sperm, detection of sperm DNA fragmentation, morphology, and membrane integrity. Nanotechnological platforms improve upon traditional methods, enhances assisted reproductive efficiency, and pioneers novel sperm selection pathways.

#### 2.3.1 Magnetic activated cell sorting (MACS)

MACS exploits magnetic fields to isolate cells by targeting phosphatidylserine (PS), a marker externalized on the membranes of apoptotic cells. In this protocol, calcium-dependent Annexin V binds to this externalized PS, allowing Annexin V-conjugated magnetic microspheres to selectively sequester apoptotic spermatozoa within a magnetic field.^59^

Compared to conventional sperm selection techniques, MACS requires smaller sample volumes and offers higher selectivity, thereby helping to reduce sperm DNA fragmentation (SDF).^60^ Sperm DNA fragmentation (SDF) and its quantitative measure, the DNA fragmentation index (DFI), serve as crucial biological markers reflecting the degree of impaired sperm DNA integrity. They are used to assess the percentage of sperm with DNA breaks within a sample.^61^ Clinical studies indicate that elevated SDF levels correlate significantly with increased miscarriage risk and poor ART outcomes.^62^ Mantravadi *et al.* reported that MACS-selected sperm in patients with high-DFI populations (DFI > 30%) improved embryo implantation rates and selected sperm with lower DNA fragmentation rates.^63^ Mateizel *et al.* compared DGC, sperm washing (SW), MACS, and sperm separation devices (SSD) to explore optimal sperm pretreatment protocols. Results indicated MACS reduced DFI by 0.27%, with varying improvements in sperm forward motility, morphological structure, and acrosome integrity index.^64^ Furthermore, Mei *et al.* found that integrating MACS with traditional DGC or SU protocols yields superior clinical pregnancy rates and chromatin maturity compared to standalone methods.^65^

Despite its utility, MACS fails to completely sequester damaged spermatozoa and may diminish both total and rapid progressive motile sperm counts. When integrated into ICSI protocols, its impact on the cumulative live birth rate (CLBR) remains clinically negligible.^66^ Consequently, large-scale randomized controlled trials are warranted to further delineate the clinical advantages of MACS within ART and firmly establish its therapeutic value (Fig. 1G).

#### 2.3.2 Microfluidic technology

Microfluidic systems constitute a nanotechnology that enables cell sorting and sample processing by controlling fluid flow within microchannels.^67^ Microfluidic technology offers multiple advantages for sperm separation: firstly, it effectively distinguishes motile from non-motile sperm, enhancing the likelihood of selecting high-quality sperm; secondly, it reduces ROS damage to sperm DNA, improving DNA integrity; thirdly, compared to traditional methods, it requires significantly less semen sample volume, with some microchannels needing only 1 milliliter of semen.^68^

Microfluidic sperm selection primarily relies on two approaches: biochemical parameter detection and kinetic parameter detection. Biochemical-level sperm screening overcomes limitations of traditional methods by detecting semen pH and sperm concentration, offering new options for patients with poor sperm motility.^68^ Mu *et al.* developed a biochemical-level, automatic-screening/separation, smart droplet-TO-hydrogel chip (BLASTO-chip) for sperm selection, for sperm selection. The chip detects pH changes induced by sperm respiratory by-products to screen biochemically active sperm with over 90% accuracy. In clinical applications, it successfully isolated viable sperm from samples containing 10% viable but 100% non-motile sperm, significantly improving fertilization rates, cleavage rates, and success rates for early-stage embryos and blastocysts^69^ (Fig. 1H-a).

Sperm selection based on kinetic parameters primarily evaluates sperm motility. Sheibak *et al.* found that sperm selected by microfluidic sperm sorters (MSS) demonstrated significantly superior motility and DFI compared to DGC and SU, while also increasing the proportion of non-apoptotic sperm. MSS yielded consistent results across diverse semen samples, demonstrating excellent universality and scalability.^70^ Zeaei *et al.* proposed a microfluidic chip that successfully separates highly motile sperm by exploiting intrinsic sperm hydrodynamics. This method can isolate over 16 000 motile sperm within 20 minutes, improving motility, motile sperm count, and DNA integrity by 45%, 20%, and 80% respectively, demonstrating strong potential for assisted reproductive applications^71^ (Fig. 1H-b).

Microfluidic technology offers significant advantages over traditional methods in screening for sperm DNA fragmentation index (DFI), motility, and structural integrity. Nevertheless, microfluidic technology confronts several unresolved challenges. Manufacturing processes for specific devices remain complex and costly, while biocompatibility requires further validation. Most devices necessitate simulation of physiological environments, involving intricate operational procedures that present considerable difficulties and demand urgent optimization. Overall, microfluidic technology offers a simple, economical, and high-throughput approach for sperm selection. Future efforts should aim to integrate it with technologies like artificial intelligence to expand its application possibilities.

## 3 Research on nanotechnology for oocyte quality enhancement and protection

Oocyte quality is a primary determinant of female reproductive potential. During ART procedures, oxidative stress induced by *in vitro* manipulation remains a significant factor limiting IVF success rates.^72^ Nanotechnology, with its efficient and targeted delivery capabilities, offers novel solutions to overcome this technical bottleneck.^73^

In promoting oocyte maturation, exosomes facilitate ovarian cell expansion and nuclear maturation by delivering natural regulatory factors.^74^ Furthermore, synthetic nanocarriers can effectively enhance oocyte quality by precisely modulating cell membrane potential and delivering artificially loaded antioxidants.^75^ In cryopreservation, the photothermal effect of nanoparticles effectively suppresses ice crystal damage, offering a safer and more efficient solution for fertility preservation.^76^ This section will explore research advances in nanotechnology for oocyte maturation, ovarian microenvironment regulation, and cryopreservation, providing an in-depth analysis of its prospects for clinical translation to offer new perspectives and approaches for reproductive medicine development.

### 3.1 Applications of nanotechnology in promoting oocyte development and maturation

Gamete quality is a critical determinant of fertilized embryo developmental potential. Oocyte quantity and quality directly influence female fertility and pregnancy outcomes.^77^ Despite significant advances in ART, *in vitro* manipulation of gametes may induce oocyte oxidative stress,^78^ thereby compromising the success rates of IVF. Achieving precise delivery of substances into germ cells without compromising their developmental potential remains a key challenge for advancing reproductive biology and optimizing ART techniques.^79^ Against this backdrop, nanotechnology demonstrates broad application potential in reproductive medicine due to its non-invasive and highly targeted characteristics.

#### 3.1.1 Extracellular vesicles (EVs)

Oocytes communicate with surrounding cells through multiple pathways to promote developmental maturation. Exosomes, as secreted vesicular structures, are present at various stages of oocyte development and maturation.^80^ Follicular fluid contains diverse exosomes whose miRNAs, proteins, lipids, and other constituents influence gene expression and participate in oocyte development regulation.^81^ Follicular fluid-derived extracellular vesicles (FF-EVs) promote oocyte maturation by delivering regulatory factors. Hung *et al.* co-cultured mouse COCs with bovine-derived FF-EVs, observing that FF-EVs induced cumulus cell migration and extracellular matrix remodeling by delivering inducers such as Ptgs2, Ptx3, and Tnfaip6, thereby supporting cumulus cell expansion.^82^ Ávila *et al.* discovered that vesicles with low progesterone levels are enriched with miRNAs regulating processes including MAPK, Hippo signaling pathways, and oocyte meiosis. These miRNAs can upregulate differentiation genes GDF9, ZP3, immune response-related genes IL6 and ARG1, and Notch signaling (HEY1)-related genes in cumulus cells. The miRNA composition of FF-EVs dynamically changes with the estrus cycle, offering a novel strategy for enhancing oocyte maturation *in vitro*.^83^

**Fig. 2:**
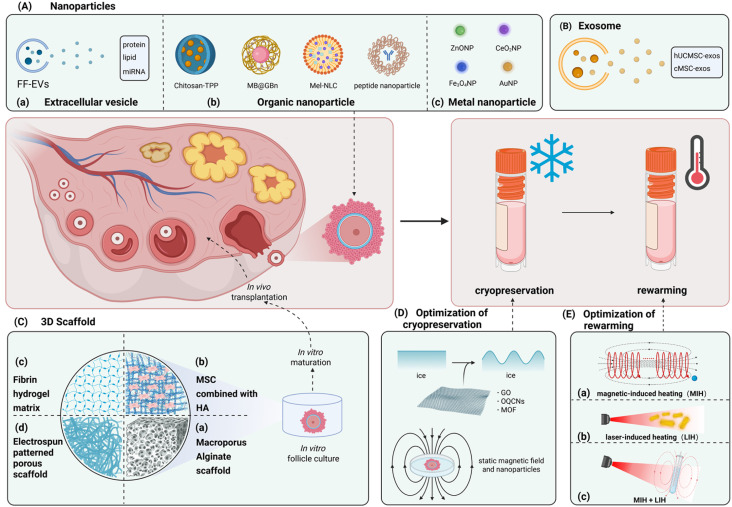
Research on nanotechnology for oocyte quality enhancement and protection. (A) Nanoparticles. (a) Extracellular vesicles. (b) Organic nanoparticles. (c) Metallic nanoparticles. (B) Exosomes. (C) 3D Scaffold. (a) Macromolecular alginate scaffolds. (b) Stem cells combined with hyaluronic acid. (c) Fibrin hydrogel scaffolds. (d) Electrospun porous scaffolds. (D) Optimization of Cryopreservation. (E) Optimization of rewarming. (a) Magnetic-induced heating (MIH). (b) Laser-induced heating (LIH). (c) MIH + LIH.

Exosomes are not only present in follicular fluid but also originate from the oviduct, uterus, vagina, and other reproductive tissues, playing crucial roles in gamete development, fertilization, and pregnancy. However, the specific markers for the isolation and purification of these exosomes require further investigation, and the large-scale acquisition of naturally sourced exosomes remains challenging. Therefore, further exploration of specific markers for various types of exosomes to expand their production scale is an urgent issue to be addressed (Fig. 2A-a).

#### 3.1.2 Organic nanoparticles

Over recent years, research into organic nanoparticles, including natural polymer scaffolds and liposomes, has attracted considerable interest. Chitosan-based nanocarriers, featuring a hydrophobic core and hydrophilic shell, effectively encapsulate hydrophobic drugs and prolong their distribution time, making them efficient drug carriers. Hashem *et al.* constructed chitosan-TPP nanoparticles to encapsulate and deliver gonadotropin-releasing hormone (GnRH). They observed that injection of this complex increased corpus luteum numbers, significantly elevated serum estradiol (E2) and progesterone (P4) concentrations, and promoted oocyte maturation.^84^ Xi H. *et al.* employed bilirubin-conjugated glycosylated chitosan (MB@GBn) as an outer layer to self-assemble endogenous bilirubin and melatonin, thereby alleviating oxidative stress during *in vitro* maturation (IVM) of oocytes.^85^ Noori *et al.* developed melatonin-loaded nanostructured lipid carriers (Mel-NLCs), validating their efficacy in enhancing oocyte maturation within an IVF environment. Mel-NLCs demonstrated a burst release of melatonin within the first two hours, followed by sustained release over 48 hours, significantly prolonging drug delivery duration compared to commercially available suspension formulations.^86^ Li *et al.* employed a peptide nanoparticle-mediated antibody transfection approach to deliver anti-Arl2 antibodies, successfully achieving specific inhibition of Arl2 in mouse oocytes. This method proves suitable for difficult-to-transfect cell types and can be extended to siRNA delivery, broadening its application scope.^87^

These organic nanoparticles promote oocyte maturation through targeted delivery of antioxidants and transfection of antibodies to regulate gene expression, serving as effective regulatory strategies in IVM to enhance ART efficacy. Numerous other polymeric scaffolds, such as poly(lactic-*co*-glycolic acid) (PLGA), hyaluronic acid, and chondroitin sulphate derivatives, have demonstrated excellent drug-carrying capacity and biocompatibility.^88,89^ Future research may develop additional polymeric nanoscale scaffolds encapsulating hormones to improve oocyte quality further. Regarding cell transfection methods, exemplified by peptide nanoparticles, regulating core genes and key proteins involved in oocyte maturation could expand their developmental potential (Fig. 2A-b).

#### 3.1.3 Metal nanoparticles

Metal nanoparticles promote follicular growth and enhance oocyte quality by modulating oxidative stress and improving oocyte structure. Fatemi *et al.* demonstrated that curcumin-loaded superparamagnetic Fe_3_O_4_ nanoparticles promoted follicular growth in a polycystic ovary mouse model. They reduced apoptosis-associated proteins while increasing survival-associated protein Bcl-2, thereby facilitating natural ovulation and pregnancy, and promoting healthy offspring.^90^ CeO_2_NPs engineered by Zhang D. M. *et al.*, incorporating alpha-lipoic acid and polyethene glycol modifications, demonstrated potent ROS scavenging capacity, reduced oocyte fragmentation, enhanced fertilization capacity and blastocyst development rates, whilst exhibiting favorable biocompatibility.^91^ Majidi *et al.*'s research indicates that combined l-carnitine and ZnONPs treatment in diabetic rats enhances antioxidant activity and significantly increases follicle count.^92^ Quintão *et al.* found ZnONPs mitigate oxidative stress damage to oocytes and increase oocyte numbers.^93^ Nguyen *et al.* indicated that gold nanoparticles (AuNPs) could enhance stability through surface modification, carry multiple payloads, and serve as IVM medium supplements to improve assisted reproductive efficiency.^94^

However, safety concerns regarding metallic nanomaterials are growing. ZnONPs show toxicity to ovarian germ cells,^95^ copper oxide nanoparticles (CuONPs) damage mitochondria and impair oocyte maturation.^96^ Furthermore, silver nanoparticles (AgNPs), titanium dioxide nanoparticles (TiO_2_NPs), and silica nanoparticles (SiO_2_NPs) may all induce abnormal hormone secretion, trigger oxidative stress, disrupt follicular development, and even cause infertility.^97^ Therefore, future efforts should focus on evaluating the safety of these nanoparticles, optimizing their performance, and exploring the feasibility of clinical applications (Fig. 2A-c).

### 3.2 Nanostrategies for optimizing the ovarian microenvironment

The ovarian microenvironment provides nutrients and signals to follicles, influencing their growth and maturation. Its composition includes the extracellular matrix (ECM), ovarian stromal cells, and ovarian stem cells.^98^ The ECM supports follicular development and capillary formation, playing a crucial role in corpus luteum formation. Ovarian stromal cells and stem cells also exert a significant influence on follicular development and functional maintenance.^99^

Pathological alterations encompass ECM fibrosis, impaired angiogenesis, and cellular senescence.^100^ Fibrosis impairs ovulation and endocrine function, stem cell depletion and senescence generate a pro-inflammatory microenvironment that compromises ovarian function.^101,102^ These changes interact synergistically to accelerate ovarian functional decline.

Nanotechnology can improve the ovarian microenvironment, with exosomes serving as natural carriers transporting multiple molecules to promote cellular repair and angiogenesis.^103^ 3D scaffold nanotechnology mimics the ovarian microenvironment, supporting follicular survival and development. Combined application holds promise as a novel approach for treating ovarian failure and improving fertility.^104^

#### 3.2.1 Exosoms

Exosomes significantly promote ovarian angiogenesis by regulating the expression of factors like VEGF. Research by Yang *et al.* demonstrated that human umbilical cord mesenchymal stem cell-derived exosomes (hUCMSC-exos), carrying VEGF-R2, MCP-1, and VEGF, can upregulate VEGF, IGF-1, and Angiogenin in mouse ovaries. They activate the PI3K/AKT pathway to exert a pro-angiogenic effect on ovarian microvasculature.^105^ Qu *et al.* investigated the impacts of hUCMSC-exos on ovarian granulosa cells in a premature ovarian failure model. They discovered that miR-126-3p contained within the exosomes could upregulate VEGF, IGF-1, and FGF, thereby promoting angiogenesis, inhibiting apoptosis, and improving ovarian function.^106^ Exosomes can also optimize the ovarian microenvironment by improving the immune microenvironment, reducing inflammation, and enhancing cellular vitality. Eslami N. *et al.* evaluated the effects of distinct exosome subpopulations (EV20K and EV110K) derived from cloned mesenchymal stem cells (cMSC-exos) on restoring function in prematurely aged ovaries. They found exosomes acted on granulosa cells and the ovarian microenvironment, reducing inflammatory factors, activating the PI3K/AKT pathway, and promoting angiogenesis.^107^ These studies show that exosomes offer practical approaches for improving the ovarian microenvironment through mechanisms like antioxidant effects and enhancement of the microenvironment (Fig. 2B).

#### 3.2.2 Three-dimensional scaffolds

3D scaffolds support follicular cells, effectively maintaining follicular structure and intercellular interactions, and are suitable for culturing large-volume human follicles.^108^ Compared to traditional 2D culture, 3D systems better mimic the *in vivo* microenvironment and preserve cell–matrix interactions.^109^ Moreover, 3D culture systems can replicate multiple processes within the microenvironment, including soluble signaling, cell migration, and tissue development.^110^ Gels, fibrin, and electrospun fibers can all serve as 3D scaffolds, recreating the natural biochemical environment of follicles within the ovary to establish an optimized microenvironment for *in vitro* culture (Fig. 2C).

Gel scaffolds provide structural support for cells and tissues, rendering them suitable for follicular culture. Scaffold design employs not only highly biocompatible materials but also facilitates nutrient diffusion and metabolic waste clearance.^111^ Felder *et al.* constructed porous alginate hydrogel scaffolds combined with bone morphogenetic protein-4 (BMP-4) self-assembled nanocomposites.^112^ Compared to conventional alginate scaffolds, this scaffold exhibits enhanced loading capacity and prolongs the release duration of BMP-4 in culture medium. *In vitro* culture results demonstrated the scaffold's successful support of follicular growth, increased follicular numbers, and promotion of follicular maturation. *In vivo* transplantation experiments further revealed the scaffold's favorable efficacy in facilitating early microenvironment vascularization (Fig. 2C-a).

The integration of stem cell technology with 3D scaffolds offers novel approaches for ovarian repair. Jiao *et al.* investigated the therapeutic efficacy of umbilical cord-derived mesenchymal stem cells (UC-MSCs) combined with hyaluronic acid (HA) gel in mice with premature ovarian failure. UC-MSCs activated the PI3K-AKT pathway *via* HGF and inhibited mTOR, while HA prolonged their retention time within the ovary. This system provides a novel strategy for MSC-based treatment of ovarian ageing^113^ (Fig. 2C-b).

With advances in materials science, smart responsive hydrogels demonstrate immense application potential. Shi *et al.* developed a novel hydrogel capable of releasing inhibitors upon RTK activation, thereby reducing mTOR activity, protecting ovarian cells, promoting microvascular formation, and delaying ageing. Its high targeting specificity and low dosage render it suitable for treating premature ovarian failure and ageing.^114^ Yang *et al.* combined hydrogels with fibrin to encapsulate nitric oxide (NO) nanoparticles within the hydrogel matrix. This approach effectively promoted vascularization in transplanted ovaries, enhancing both the quantity and quality of follicles within the graft. It successfully supported the *in vitro* fertilization process and blastocyst formation of oocytes^115^ (Fig. 2C-c).

Electrospinning, a technique employing high-voltage electric fields to produce ultrafine fibers, demonstrates potential in ovarian tissue engineering by improving follicular adhesion and survival.^116^ This method mimics the ovarian extracellular matrix, facilitates cell interactions, and provides an appropriate microenvironment for cells. L. Liverani *et al.* employed electrospinning to fabricate PCL/gelatin scaffolds with nanoscale pores, exhibiting superior three-dimensional architecture. These scaffolds facilitate follicular attachment, growth, and proliferation, thereby boosting follicular survival rates and promoting vascularization to repair damaged ovaries structures^117^ (Fig. 2C-d).

The successful application of the matrix above provides a research direction for next-generation advanced follicular culture systems. Nevertheless, fibrin degrades readily *in vitro*, potentially compromising the scaffold's capacity to support follicular development. Enhancing its biostability thus remains an urgent technical difficulty.^118^ Existing electrospinning techniques cannot precisely control fiber diameter, leaving the precise construction of nanofibers an unresolved challenge.^119^ Furthermore, their biodegradability and biosafety require further clarification. Selecting suitable materials to ensure degradation rates and safety remains a considerable undertaking.

### 3.3 Nanoprotective technologies for oocyte cryopreservation

In recent years, demand for fertility preservation has significantly increased due to malignant tumors, benign conditions, societal factors, and age-related fertility decline.^120^ Currently, embryo cryopreservation, oocyte cryopreservation following ovarian stimulation, and ovarian tissue cryopreservation are the three mainstream fertility preservation strategies recommended by the American Society for Reproductive Medicine.^121^ Among these, oocyte cryopreservation is increasingly sought as societal acceptance and insurance coverage expand.^122^ Nanotechnology shows promise in this field by enabling dynamic regulation of cryopreservation parameters, enhancing efficiency and reducing cryoinjury.^123^ Through optimized material properties, it can improve the cryoprotective microenvironment.^124^

Oocyte cryopreservation achieves long-term preservation of female fertility by suppressing cellular metabolism and biochemical reactions under ultra-low temperatures.^125^ Commonly employed cryopreservation methods include programmed slow-freezing and vitrification. Slow-freezing techniques control cooling rates to minimize intracellular and extracellular ice crystal formation, although improper rates may cause cellular damage.^126^ In contrast, vitrification employs extremely rapid cooling to form an amorphous glassy state, effectively preventing ice crystal damage and is widely recognized as one of the most efficient cryopreservation methods currently available.^127^ Nevertheless, this technique still faces challenges in controlling ice crystal formation and optimizing the thawing process.

#### 3.3.1 Reducing ice crystal formation

Minimizing ice crystal formation is a critical step in the cryopreservation process. Crystallization not only causes mechanical damage to cell membranes but may also disrupt intracellular macromolecular structures. Research indicates that effective control of ice crystal formation enhances oocyte survival rates and embryo developmental capacity,^128^ thereby mitigating adverse effects on post-thaw IVF outcomes. Consequently, developing nanomaterials to inhibit ice crystal formation has become a research focus. Graphene oxide (GO) is a novel material exhibiting excellent ice crystal suppression capabilities. H. Geng *et al.* discovered that GO binds to the basal or prismatic faces of ice crystals, causing surface curvature that lowers the freezing point and inhibits crystal growth. Molecular dynamics simulations reveal that carboxyl groups on GO surfaces form more hydrogen bonds with ice than with liquid water, providing a reliable molecular mechanism for controlling ice crystal formation.^129^ Furthermore, oxide quasi-carbon nitride quantum dots (OQCNs) and zirconium-based metal–organic framework (MOF) nanoparticles have also been demonstrated to effectively inhibit ice crystals and enhance cell survival rates.^130^ OQCNs exhibit thermal hysteresis effects and morphoregulatory capabilities in ice, whilst MOFs demonstrate application value in cellular cryopreservation by inhibiting ice recrystallization and promoting ice crystal melting. Baniasadi *et al.* investigated the effects of static magnetic fields (SMF) and iron oxide nanoparticles on the vitrification of cumulus oocyte complexes (COCs). Their findings indicate that the combination of SMF and nanoparticles restored the normal expression of key genes, such as Cdx2, effectively mitigating cryoinjury and significantly enhancing the developmental potential and blastocyst formation rates of the COCs^131^ (Fig. 2D).

#### 3.3.2 Optimization of the thawing process

Thawing rate directly impacts cell survival and functional recovery. To enhance thawing efficiency and uniformity, researchers have developed and applied multiple nano-assisted thawing techniques.

In magnetothermal rewarming, J. Pan *et al.* designed an electromagnetic resonance rewarming system incorporating magnetic nanoparticles (MNPs) to enhance energy absorption and conversion. This achieved rewarming rates exceeding 200 °C min^−1^ and successfully rewarmed milliliter-scale samples. The use of extremely low MNP concentrations (0.1 mg per mL Fe) suggests potential for its application in tissue and organ preservation.^132^ A. Ito *et al.* achieved uniform rapid rewarming using MNPs, significantly improving survival rates in large-volume specimens^133^ (Fig. 2E-a).

Photothermal rewarming technology employs the photothermal effect of nanoparticles to elevate temperature, preventing ice crystal reformation rapidly. K. Khosla *et al.* utilized gold nanorods and laser pulses to rewarm zebrafish embryos, achieving survival rates comparable to unfrozen controls, with some individuals developing into reproductive adults.^134^ Y. Hou *et al.* developed a photothermal rewarming system based on liquid metal nanoparticles, significantly enhancing rewarming rates and tissue survival under low cryoprotectant concentrations while demonstrating excellent biocompatibility^135^ (Fig. 2E-b).

Research integrating magnetic-thermal and photothermal advantages has enhanced rewarming performance. Tian *et al.* developed a low-toxicity vitrification method utilizing the dual thermal effects of GO and Fe_3_O_4_ nanoparticles, enabling follicular precursors to retain morphology and function post-rewarming, ultimately yielding healthy offspring *via in vitro* fertilization.^136^ Karimi *et al.* employed PEG-modified silica-coated iron oxide nanoparticles to thaw ovarian tissue within an alternating magnetic field. This approach markedly improved antioxidant markers, reduced apoptosis, and restored follicular development and associated gene expression to levels approaching those of fresh tissue^137^ (Fig. 2E-c).

The integration of nanomaterials with novel thawing techniques enhances oocyte cryopreservation efficacy. However, materials such as GO, OQCNs, Fe_3_O_4_ and MOFs may exhibit cytotoxicity at high concentrations, necessitating further investigation into their biocompatibility, stability, and scalable production feasibility. Furthermore, achieving uniformity and rate control during thawing, particularly in large-volume samples, remains a technical challenge.

Antioxidant strategies based on nanotechnology can significantly mitigate oxidative damage, improving the survival rate and developmental capacity of cryopreserved oocytes. Future work should elucidate their molecular mechanisms, optimize dosages and delivery systems, and evaluate the safety of nanomaterials. Advancing the integration of nanotechnology with antioxidant strategies will facilitate the establishment of more efficient and secure oocyte cryopreservation systems.

## 4 Applications of nanotechnology in diagnosing and treating reproductive system disorders

The management of gynecological disorders, particularly those impacting reproductive function, presents substantial clinical challenges.^138^ These stem from pathological complexity, limitations in targeted therapies, and the invasive nature of many procedures.^139^ In this context, nanotechnology has emerged as a transformative frontier in biomedical science. It offers novel solutions through enhanced drug delivery, improved imaging, and innovative regenerative strategies.^140^

Nanotechnology demonstrates distinct advantages. By utilizing platforms such as engineered extracellular vesicles and composite nanofiber scaffolds,^141^ nanomaterials enable precise delivery of bioactive molecules to promote the restoration of ovarian function. Hydrogel systems can achieve minimally invasive repair of fallopian tube injuries.^142^ In the context of endometriosis, nanotechnology further advances the development of non-invasive diagnostic techniques and targeted therapeutic strategies, such as intervening in disease progression through the modulation of glucose metabolism, attenuation of oxidative stress, and inhibition of angiogenesis.^143,144^ These nano-based strategies offer more precise and minimally invasive diagnostic and therapeutic options for gynecological diseases. When integrated with existing clinical technologies, they hold significant potential for markedly improving fertility preservation and disease treatment outcomes.

### 4.1 Nanotechnology-based therapies for ovarian dysfunction and premature ovarian failure

Premature ovarian insufficiency (POI) denotes ovarian dysfunction occurring before age 40, characterized by menstrual irregularities (amenorrhea or oligomenorrhoea) accompanied by elevated gonadotropin (Gn) levels and fluctuating estrogen decline. Unlike natural menopause in women, POI not only impacts fertility, psychological well-being, and quality of life but also poses significant risks to cardiovascular, urogenital, musculoskeletal, and cognitive health. Its etiology encompasses genetic, immunological, infectious, environmental, and iatrogenic factors, though precise mechanisms remain unclear. To date, no definitive method exists to restore ovarian function. Premature ovarian failure (POF) represents the terminal stage of POI primarily manifesting as amenorrhea before age 40, elevated Gn levels, and reduced estrogen levels, potentially accompanied by varying degrees of hypoestrogenic symptoms.^145^

**Fig. 3:**
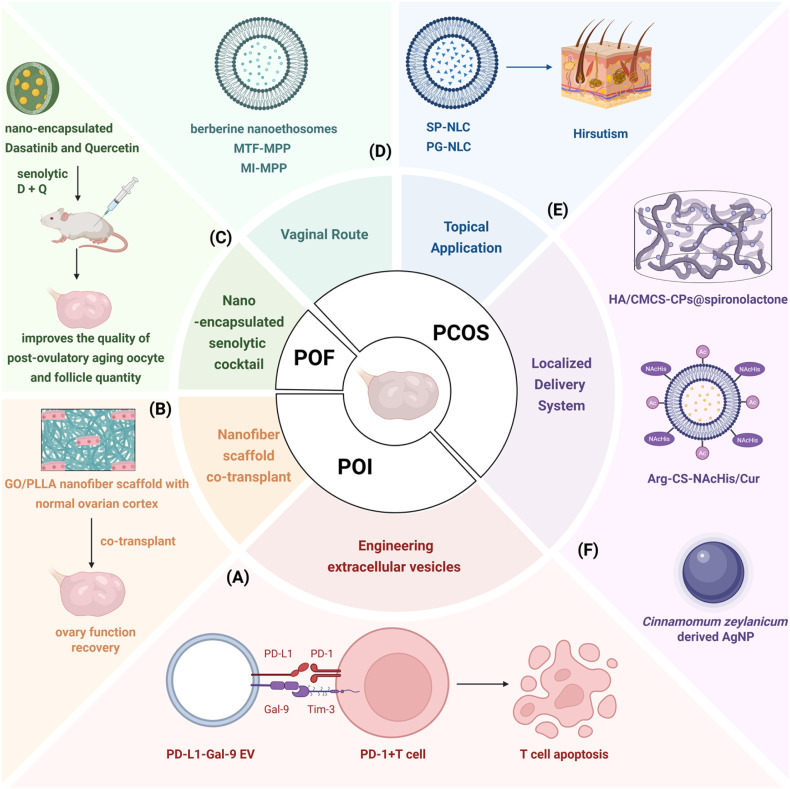
Nanotherapeutic approaches for ovarian insufficiency, premature ovarian failure, and polycystic ovary syndrome. (A) Engineering extracellular vesicles. (B) Nanofiber scaffold co-transplant. (C) Nano-encapsulated senolytic cocktail. (D) Vaginal route. (E) Topical application. (F) Localized delivery system.

Both POI and POF represent significant causes of female infertility. Present-day clinical interventions, including hormone replacement therapy, ovulation induction, and stem cell transplantation, face limitations including inefficient drug delivery, inadequate targeting, and poor cell survival rates. In recent years, nanotechnology has demonstrated potential application value in ovarian function restoration due to its characteristics of precise delivery, controlled release, and multifunctional integration. For instance, Zhou *et al.* targeted immune pathways in POI pathogenesis by constructing a bioengineered nanoplatform using engineered extracellular vesicles as scaffolds to deliver PD-L1 and Gal-9. This approach inhibited ovarian autoimmunity and restored serum anti-Müllerian hormone (AMH) levels in a POI model, thereby halting disease progression and preserving ovarian function^146^ (Fig. 3A).

L. Yan *et al.* innovatively constructed graphene oxide/polylactic acid (GO/PLLA) composite nanofiber scaffolds. Using a mouse POF model, they investigated the efficacy of this nanomaterial encapsulating normal ovarian tissue for combined transplantation. Experiments demonstrated that co-transplantation of GO/PLLA material with ovarian tissue increased anti-Müllerian hormone (AMH) and estradiol (E2) levels while reducing follicle-stimulating hormone (FSH) levels. It also increased total follicle counts on both transplanted and non-transplanted sides. This innovative approach offers a novel strategy for preserving female fertility^147^ (Fig. 3B).

Guan *et al.* encapsulated the anti-ageing drugs dasatinib and quercetin within nanoparticles to enhance their water solubility. In a cyclophosphamide-induced POF model, the nano-encapsulated senolytic D + Q cocktail effectively improved the quality of aged oocytes and increased follicular numbers. Concurrently, it significantly reduced ROS levels, thereby mitigating DNA damage and apoptosis. This nanotechnology holds promise for improving POF and ART outcomes^148^ (Fig. 3C).

Although clinical research on nanotherapeutics for POI/POF remains in its infancy, nanotechnology explorations in other reproductive fields (*e.g.*, endometriosis, polycystic ovary syndrome) have been extensively documented. Consequently, nanotherapeutic approaches for POI and POF are considered to possess considerable translational potential.^149^

### 4.2 Advances in nanomedicine for treating polycystic ovary syndrome

Polycystic ovary syndrome (PCOS) is a prevalent gynecological endocrine disorder characterized clinically by hyperandrogenism and ovulatory dysfunction. Conventional pharmacological treatments like clomiphene citrate and metformin show limited effectiveness due to poor drug targeting and significant side effects, highlighting the need for safer and more efficient therapies strategies.^150^ In recent years, nanotechnology has demonstrated immense potential in the biomedical field due to its unique physicochemical properties and delivery advantages.

Hydrogel nanoparticles offer significant advantages in treating polycystic ovary syndrome, owing to their unique three-dimensional network structure, which provides excellent drug-loading capacity, favorable targeting properties, outstanding biocompatibility, and degradability. Usulkar *et al.* enhanced the efficacy of nano-nanocapsules for treating PCOS by developing, evaluating, and refining a vaginal *in situ* gel containing, thus increasing drug penetration, achieve controlled release kinetics, and mitigate adverse reactions associated with oral berberine administration.^151^ Farooq *et al.* designed a nano-biphasic formulation comprising metformin-loaded mucus-penetrating nanoparticles (MTF-MPP) and inositol-loaded mucus-penetrating particles (MI-MPP). Administered vaginally by mixing the biphasic formulation into a carbomer gel, *in vivo* studies compared this approach with conventional vaginal gels. This formulation significantly reduced ovarian weight while demonstrating non-irritant, safe therapeutic effects, thereby offering a promising strategy for vaginal drug delivery^152^ (Fig. 3D).

Conditions like PCOS, which are characterized by high levels of male hormones, often present with excessive hair growth. Amer *et al.* investigated the follicle-targeting effects of topical spironolactone (SP) *versus* progestogen-loaded (PG) nanostructured lipid carriers (NLCs) on hirsutism. And prepared SP-NLC and PG-NLC topical hydrogels to investigate their pharmacological effects on letrozole-induced PCOS in rats. Topical application of SP or PG nanogel resulted in significantly reduced hair follicle diameter and density. The impact of these locally administered nanostructured lipid carriers on hirsutism offers a potential therapeutic approach for PCOS^153^ (Fig. 3E).

Wang *et al.* synthesized two novel coordination polymers (CPs) containing Co(ii), subsequently encapsulating these CPs within hyaluronic acid (HA) and carboxymethyl chitosan (CMCS) hydrogels and ultimately yielding two types of metal gel particles carrying spironolactone (HA/CMCS-CPs@spironolactone). An *in vitro* PCOS cell model was established, and PCOS cells were treated with the metal gel particles. Levels of malondialdehyde (MDA), a key indicator of oxidative stress, were measured. Results demonstrated that both metal gel particles reduced MDA levels in a dose-dependent manner, offering a promising therapeutic option for PCOS.^154^ Raja *et al.* developed curcumin (Cur)-encapsulated chitosan (Arg-CS-NAcHis/Cur) nanoparticles modified with arginine (Arg) and *N*-acetylhistidine (NAcHis). *In vitro* drug release experiments demonstrated sustained release from these nanoparticles, while cytotoxicity and cellular uptake studies showed superior efficacy compared to free curcumin. Biochemical and histopathological analyses confirmed the nanoparticle's positive effect on symptom recovery in PCOS rats.^155^ Alwan *et al.* experimentally assessed the effects of Cinnamomum zeylanicum (CZ)-derived AgNPs on inflammatory cytokines in PCOS rats. They found that CZ-derived AgNPs may exert anti-inflammatory effects by reducing cytokine concentrations of TNF-α, IL-6, and IL-18 in PCOS rats^156^ (Fig. 3F).

### 4.3 Advances in nanotechnology for diagnosis and treatment of tubal diseases

Tubal diseases account for 25–35% of female infertility. As the site of fertilization is located within the female fallopian tubes, obstruction or pathological changes in this region inevitably reduce conception rates and may even cause infertility.^157^ Consequently, diagnosing tubal patency and treating associated diseases holds significant value in infertility management.

**Fig. 4:**
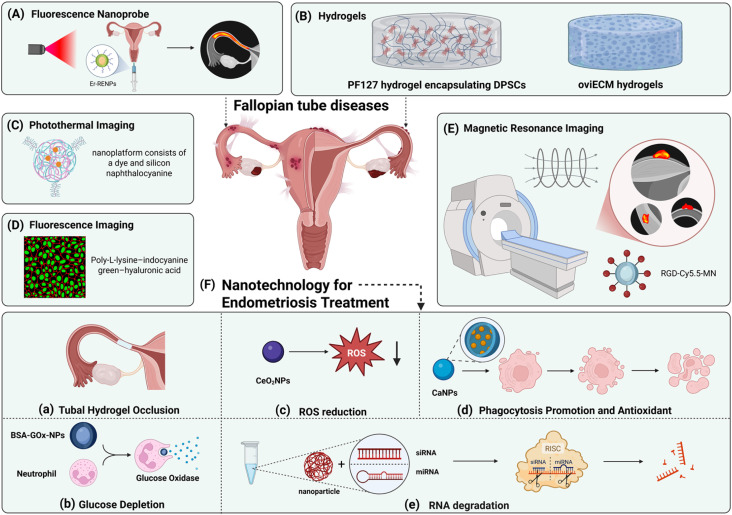
Nanotherapeutic approaches for tubal disease and endometriosis. (A) Fluorescence nanoprobe. (B) Hydrogels. (C) Photothermal imaging. (D) Fluorescence imaging. (E) Magnetic resonance imaging. (F) Nanotherapy for endometriosis. (a) Tubal hydrogel occlusion. (b) Glucose depletion. (c) ROS reduction. (d) Phagocytosis promotion and antioxidant. (e) RNA degradation.

Currently, the primary clinical method for assessing fallopian tube patency is hysterosalpingography, which carries disadvantages including radiation exposure and a high false-positive rate.^158^ Nanotechnology offers distinct advantages in non-invasive diagnostics, featuring excellent tissue penetration and low signal-to-noise ratios. Duan *et al.* synthesized a rare-earth erbium-based nanoprobe (Er-RENPs) with superior near-infrared II (NIR-II) fluorescence properties using NIR-II fluorescence imaging. This probe exhibits good biocompatibility and can clearly delineate fallopian tube contours, enabling diagnosis of narrowed, obstructed tubes and hydrosalpinx. It demonstrates significant application potential in diagnosing fallopian tube disorders^159^ (Fig. 4A).

Fallopian tube damage constitutes a significant factor in infertility. Common clinical treatments for tubal disorders include antibiotic therapy and laparoscopic surgery, which may lead to varying degrees of tubal adhesions and antibiotic resistance.^160^ Although nanotechnology for treating tubal infertility remains in its infancy, it demonstrates formidable application potential. Current research primarily focuses on hydrogel systems. For instance, Luo *et al.* encapsulated dental pulp stem cells within hydrogels and performed *in situ* transplantation at sites of tubal injury to facilitate repair and regeneration.^161^ Francés-Herrero and colleagues pursued an alternative approach by creating a fallopian tube-specific extracellular matrix (oviECM) hydrogel. Its rich bioactive components perfectly mimic the natural environment, thereby optimizing the developmental pathways for cultured embryos^162^ (Fig. 4B).

### 4.4 Nanotechnology for diagnosis and imaging of endometriosis

Endometriosis refers to the growth, infiltration, and recurrent bleeding of endometrial tissue (glands and stroma) outside the uterine cavity lining and myometrium. This can form nodules and masses, causing pain, infertility, and other complications.^163^ Endometriosis ranks among the most prevalent conditions affecting women of reproductive age, with an incidence rate of approximately 10–15%. However, its prevalence rises to 20–25% among women experiencing infertility, exhibiting an upward trend.

Diagnosis based on visual laparoscopy remains the gold standard for identifying and staging endometriosis. Laparoscopy is an invasive procedure; while relatively safe, surgical risks cannot be overlooked, including intraoperative hemorrhage, wound infection, and surrounding tissue adhesions. Even during surgery, the coloration of endometriotic lesions can present in multiple patterns, making intraoperative differentiation from normal tissue challenging. Thus, nanotechnology presents a promising solution for enhancing the intraoperative ability of gynecologists and surgeons to differentiate between benign and malignant tissues. Taratula *et al.* designed nanoparticles composed of the fluorescent dye naphthocyanine, which activate upon internalization by endometriotic cells, emitting a fluorescent signal to distinguish pathological from normal tissue.^164^ These nanoparticles can also utilize the photothermal effect to interact with near-infrared light, enabling cellular ablation and thus achieving combined diagnostic and therapeutic objectives (Fig. 4C).

Marquardt *et al.* developed a synthetic multimodal imaging technique utilizing fluorescein isothiocyanate gold nanoparticles to label endometriosis-like lesions.^165^ Quanjie Lv *et al.* employed a poly-l-lysine-indocyanine green-hyaluronic acid nanoparticle formulation. By enhancing fluorescence photostability and antioxidant capacity, alongside low permeability and lesion surface retention properties, this approach provides novel experimental tools for investigating endometriosis and its associated infertility development^166^ (Fig. 4D). Talebloo *et al.* successfully detected lesions in an endometriosis mouse model using cRGD-peptide-conjugated nanoparticles and magnetic resonance imaging technology. This technique enables *in vivo* photoacoustic imaging detection of gold-labelled lesions in the preclinical stage, with labelled tissues readily excised *via* fluorescence dissecting microscopy. It offers novel experimental approaches for studying and understanding the development and progression of endometriosis and associated infertility^167^ (Fig. 4E).

### 4.5 Advances in nanotechnology for endometriosis treatment

Current treatment modalities for endometriosis primarily encompass pharmacological interventions, surgical procedures, and ART. Pharmacological approaches predominantly involve hormonal agents and non-steroidal anti-inflammatory drugs (NSAIDs). While hormonal therapies may alleviate symptoms and control lesions by suppressing ovarian hormone secretion, long-term use carries risks of osteoporosis and mood fluctuations. Crucially, these treatments fail to address the underlying pathology, with lesions often recurring upon discontinuation. NSAIDs merely alleviate pain without significantly affecting the lesions themselves, whilst prolonged use may cause gastrointestinal discomfort or other side effects.^168^ Removing or destroying ectopic endometrial tissue through surgery can help, but it also carries risks of complications like infection and scarring, and doesn't guarantee that the condition won't come back. Patients with severe endometriosis may undergo a total hysterectomy. This irreversible procedure significantly impacts physiological function and psychological well-being, and is unsuitable for women wishing to preserve fertility.^169^ For people with endometriosis and infertility, ART like IVF offer the best solution. However, these techniques involve complex procedures, high costs, and success rates influenced by multiple factors.^170^

Multiple studies indicate that nanotechnology may emerge as a novel therapeutic option for endometriosis. Among the various proposed etiologies, the retrograde menstrual implantation theory is widely accepted. Anthis *et al.* proposed a reversible contraceptive method by implanting a hydrogel system within a human-scale uterine model.^171^ This hydrogel system comprises two distinct acrylamide polymers crosslinked with either the photolabile molecule poly(ethylene glycol) di-photodegradable acrylate (PEGdiPDA) or the disulphide crosslinker *N*,*N*′-bis(acryloyl)cystamine (BAC), forming a hydrogel. The hydrogel system mechanically obstructs the fallopian tubes, whilst its biocompatibility was validated in a porcine model, enabling reversible sterilization and offering potential treatment for endometriosis (Fig. 4F-a).

Hormonal therapy remains the standard pharmacological treatment for endometriosis, yet hormones inevitably carry numerous side effects and cause significant bodily trauma. There is an urgent need to develop specific therapeutics for endometriosis. Capitalizing on two characteristics—the persistent recruitment of neutrophils to ectopic lesions and the high glucose uptake of ectopic cells—Zhu *et al.* designed bovine serum albumin nanoparticles (BSA-GOx-NPs) loaded with glucose oxidase. These nanoparticles are delivered to ectopic lesions in a neutrophil-dependent manner with high specificity. They consume glucose to induce apoptosis in ectopic lesions, demonstrating efficacy against both acute and chronic inflammation^172^ (Fig. 4F-b).

Numerous studies indicate that oxidative stress is implicated in the pathogenesis of various diseases, including tumors, endometriosis, and certain cardiovascular and cerebrovascular disorders. In endometriosis patients, research demonstrates elevated oxidative stress levels, manifested as increased ROS production and diminished antioxidant capacity. These alterations not only promote the proliferation and migration of ectopic endometrial cells but also trigger local inflammatory responses, exacerbate pain, and impair reproductive function. Chaudhury *et al.* demonstrated that cerium oxide nanoparticles alleviate endometrial lesions induced in mouse models by reducing oxidative stress and inhibiting angiogenesis. Furthermore, these nanoparticles protect oocytes from endometriosis effects^173^ (Fig. 4F-c). Sun *et al.* prepared acid-sensitive calcium carbonate nanoparticles (CaNP) doped with BML-111 (BML@CaNP). BML@CaNP enhances macrophage phagocytosis in a calcium ion-dose-dependent manner. Concurrently, the calcium carbonate nanoparticles synergistically exert anti-inflammatory effects with the endogenous pro-resolving lipid mediator Lipoxin A4 (LXA4), primarily through BML-111's role as a Lipoxin agonist^174^ (Fig. 4F-d).

Gene therapy demonstrates unique advantages in treating endometriosis, particularly regarding targeting precision, personalization, and sustained efficacy. Small interfering RNA (siRNA), as a gene therapy modality, can selectively silence any gene by interfering with mRNA expression. However, siRNA is a negatively charged macromolecule that struggles to traverse cell membranes; nanoparticle encapsulation technology overcomes this challenge. K. Kiisholts *et al.* discovered that RGD1-R6 peptide carriers can polymerize with siRNA to form stable nanoparticles. This nanoparticle complex was surgically administered in rat models to induce RNAi-mediated silencing of the VEGFA gene, thereby exerting anti-angiogenic effects.^175^ MiRNAs play crucial roles in gene expression and are highly correlated with the pathogenesis of certain diseases. S. Chaichian *et al.* developed poly(lactic-*co*-glycolic acid) copolymer nanoparticles (PLGA-NPs) to deliver miRNA-503 to endometriotic cyst stromal cells, thereby suppressing endometriotic cell proliferation and enhancing apoptosis^176^ (Fig. 4F-e).

Nanoparticle therapy demonstrates promising prospects in the intervention of endometriosis, offering potential as a significant future therapeutic strategy for this condition.

## 5 Translational challenges and future perspectives

Despite the rapid expansion of nanotechnology in reproductive medicine, the translation of these approaches into clinical practice remains limited. Compared with other biomedical fields, reproductive medicine imposes uniquely stringent requirements, as interventions may affect not only somatic tissues but also gametes, embryos, pregnancy outcomes, and potentially the health of future generations. Therefore, in addition to demonstrating therapeutic efficacy, nanoplatforms must meet higher standards of safety, reproducibility, and long-term biological compatibility.

### 5.1 Clinical translation potential of current nanotechnologies

Among the diverse nanotechnologies discussed in this review, their translational readiness varies substantially. *Ex vivo* approaches, particularly those integrated into ART workflows, are relatively closer to clinical application. Microfluidic sperm selection systems and MACS, for example, have already been explored in clinical or laboratory settings to enrich sperm populations with improved motility and reduced DNA fragmentation. Because these strategies operate outside the human body, systemic exposure is minimized, which may facilitate regulatory approval. However, their impact on clinically meaningful endpoints, such as cumulative live birth rates and long-term offspring outcomes, still requires further validation.

Nano-enabled cryopreservation systems and antioxidant nanomaterials also show translational promise in the context of gamete and embryo handling. By reducing oxidative stress and cryoinjury, these approaches may improve fertilization efficiency and embryo developmental competence. Nevertheless, uncertainties remain regarding optimal dosing, nanoparticle retention, and potential effects on early embryogenesis.

In contrast, most *in vivo* nanotherapeutic strategies, including nanoparticle-based drug delivery for ovarian dysfunction, PCOS, endometriosis, and tubal disease, are still largely confined to preclinical studies. Although these systems offer advantages such as targeted delivery, controlled release, and improved bioavailability, their clinical translation is constrained by challenges related to biodistribution, pharmacokinetics, interspecies variability, and safety in the reproductive context.

Biologically derived nanoplatforms, such as EVs, represent a promising emerging direction due to their intrinsic biocompatibility and ability to mediate intercellular communication. However, their translation is currently limited by difficulties in large-scale production, heterogeneity, and lack of standardized characterization methods.

### 5.2 Reproductive safety and long-term risk assessment

Safety considerations are particularly critical in reproductive nanomedicine. Unlike conventional therapeutic areas, where short-term efficacy and toxicity are often the primary concerns, reproductive applications require comprehensive evaluation across multiple biological levels.

At the gonadal level, nanoparticles may interact with spermatogenic cells, oocytes, and supporting cells such as Sertoli and granulosa cells, potentially affecting gametogenesis and hormone regulation. At the cellular and subcellular levels, nanoparticle-induced oxidative stress, mitochondrial dysfunction, DNA damage, and epigenetic alterations may compromise gamete quality.

At the embryonic level, exposure to nanomaterials during fertilization or early development raises concerns regarding embryotoxicity, including impaired cleavage, abnormal blastocyst formation, and altered implantation potential. In addition, certain nanomaterials may cross biological barriers, including the blood–testis barrier and placental barrier, thereby posing risks to fetal development.

Importantly, the possibility of transgenerational effects should not be overlooked. Epigenetic modifications or genetic damage induced by nanomaterials may theoretically be transmitted to offspring, although current evidence remains limited and largely derived from animal studies.

Taken together, future studies should incorporate systematic reproductive safety evaluation frameworks, including assessments of gamete integrity, embryo development, pregnancy outcomes, offspring health, and long-term follow-up. Establishing standardized testing protocols will be essential for advancing the safe application of nanotechnologies in reproductive medicine.

### 5.3 Manufacturing, scalability, and regulatory considerations

In addition to biological challenges, the translation of nanotechnologies into clinical practice is also constrained by manufacturing and regulatory barriers. One major issue is the reproducibility of nanomaterials. Variations in particle size, surface charge, composition, and loading efficiency between batches can significantly influence biological performance and safety profiles.

Scalable production under good manufacturing practice conditions remains difficult for many nanoplatforms, particularly for complex systems such as multifunctional nanoparticles and extracellular vesicles. Processes such as purification, sterilization, and storage may further alter nanoparticle properties, affecting stability and efficacy.

Quality control is another critical challenge. Comprehensive characterization, including physicochemical properties, endotoxin levels, residual solvents, and degradation products, is required to ensure safety and consistency. For reproductive applications, additional considerations such as sterility, absence of reproductive toxicity, and compatibility with ART procedures are particularly important.

Regulatory pathways for reproductive nanomedicine are also more stringent compared with many other therapeutic areas. Because these interventions may influence not only patients but also embryos and offspring, regulatory agencies are likely to require extensive preclinical data, including long-term reproductive toxicity and developmental studies.

### 5.4 Future perspectives

Future research in reproductive nanomedicine should move beyond proof-of-concept studies toward more standardized, mechanism-driven, and translationally oriented investigations.

First, rational design of nanoplatforms based on structure–activity relationships, including particle size, surface functionalization, and controlled release behavior, will be essential for optimizing efficacy and safety. Second, more physiologically relevant models, including organoids and advanced *in vitro* reproductive systems, may help bridge the gap between animal studies and human applications.

In addition to these advances, emerging biomimetic approaches inspired by natural antimicrobial systems are gaining increasing attention. In particular, antimicrobial peptides (AMPs), derived from innate host defense mechanisms, exhibit broad-spectrum antimicrobial activity, rapid bactericidal effects, and a relatively low propensity for inducing resistance.

Recent advances have demonstrated that these peptides can be engineered or integrated with nanomaterials and biomaterial scaffolds to enhance their stability, targeting capability, and controlled release behavior.^177–179^ Such hybrid systems combine the advantages of nanotechnology and peptide-based therapeutics, offering promising opportunities for addressing reproductive tract infections and inflammation.

These developments suggest that AMP-inspired nanostructures may serve as a complementary direction to conventional nanoplatforms, further broadening the design space for next-generation reproductive nanomedicine.

To facilitate clinical translation, well-designed preclinical studies with standardized outcome measures, followed by carefully controlled clinical trials, will be critical for evaluating therapeutic efficacy and safety. Particular emphasis should be placed on long-term reproductive outcomes and offspring health.

Finally, interdisciplinary collaboration among materials scientists, reproductive biologists, clinicians, and regulatory experts will be crucial for accelerating the translation of nanotechnology from bench to bedside. While significant challenges remain, continued progress in nanomaterial design, safety evaluation, and manufacturing technologies is expected to gradually enable the integration of nanotechnology into clinical reproductive medicine.

## 6 Conclusion

This review systematically underscores the transformative potential of nanotechnology as a frontier platform for addressing global infertility challenges. By facilitating precise antioxidant protection, targeted delivery of bioactive molecules, gene regulation, and high-throughput sperm screening, nanotechnology offers a paradigm shift to circumvent the intrinsic limitations of current ART—notably, the low efficacy in compromised gametes, cryoinjury susceptibility, and the lack of disease-specific therapeutics.

In the realm of male reproductive health, nanomaterials such as CeO_2_NPs and SeNPs have been shown to significantly augment sperm motility, morphology, and DNA integrity by mimicking enzymatic activity to scavenge ROS. Furthermore, delivery systems based on exosomes or synthetic nanocarriers provide precise interventions for sperm dysfunction arising from genetic defects (*e.g.*, Dmc1 or Pin1 deficiency) or acquired pathologies like varicocele. Concurrently, the integration of magnetic nanoparticles with microfluidics has propelled sperm sorting toward higher efficiency and reduced cellular damage. In female reproductive health, nanotechnology applications are equally diverse: stem cell-derived or follicular fluid exosomes effectively promote cumulus cell expansion and nuclear maturation. Similarly, chitosan- and liposome-based carriers successfully deliver agents such as melatonin and retinoic acid, thereby attenuating oxidative stress during IVM. Moreover, the capacity of nanomaterials to inhibit ice crystal formation and facilitate rapid, uniform rewarming significantly bolsters the efficiency of oocyte vitrification. Beyond therapeutics, nanotechnology demonstrates exceptional dual value in theranostics. Specifically, nanoprobe-based modalities, including NIR-II fluorescence and photoacoustic imaging, enable non-invasive, high-precision detection of pathologies such as endometriosis and hydrosalpinx. Meanwhile, nanocarrier systems—ranging from hydrogel nanoparticles to biomimetic vesicles—provide novel, low-toxicity therapeutic strategies for complex disorders like POI and PCOS by optimizing drug pharmacokinetics and targeting mechanisms.

Despite these advances, translating nanotechnology from bench to bedside is impeded by significant hurdles. First, biosafety remains a primary concern; specific metallic nanoparticles (*e.g.*, AgNPs and ZnONPs) exhibit cytotoxicity toward germ cells, necessitating comprehensive longitudinal evaluations of their *in vivo* metabolism, accumulation, and immunogenicity. Second, the lack of standardization and scalability presents a major bottleneck. Establishing large-scale, high-purity production processes for exosomes and complex nanomedicines is critical to guaranteeing quality control and batch consistency. Finally, clinical translational evidence is scarce. As most data are derived from animal models, rigorous clinical trials are imperative to validate safety and efficacy in humans, alongside the establishment of robust ethical and regulatory frameworks.

Looking forward, the evolution of nanobiomedicine in reproduction should prioritize four key directions: firstly, material innovation. Designing novel nanomaterials with enhanced biocompatibility, biodegradability, and stimuli-responsive properties (*e.g.*, pH- or enzyme-triggered release) to minimize off-target toxicity; secondly, mechanistic elucidation. Utilizing multi-omics and advanced imaging to decode the molecular interactions between nanomaterials, germ cells, and the reproductive microenvironment, thereby laying a theoretical foundation for rational design; thirdly, technological convergence. Integrating nanotechnology with artificial intelligence, CRISPR gene editing, and organ-on-a-chip platforms to construct high-fidelity *in vivo*-mimetic models and intelligent therapeutic systems; fourthly, clinical translation. Fostering interdisciplinary collaboration to initiate exploratory clinical studies targeting specific indications—such as idiopathic infertility and POI—while concurrently establishing standardized protocols for production and safety assessment.

In summary, nanotechnology holds the promise to revolutionize the prevention, diagnosis, and treatment of reproductive disorders. Through sustained multidisciplinary innovation and prudent translation, this frontier technology may ultimately provide safer, more effective, and personalized fertility solutions for millions of couples worldwide.

## Author contributions

Xu Wen: conceptualization, formal analysis, investigation, writing – original draft; Zhiyan Wang: writing, visualization, validation, data curation; Jiahui Lin: visualization, validation, data curation; Longjie Li: material construction, analysis; Haiyun Wang: validation; Pei Liu: investigation; Hao Hu: visualization; Chao He: resources; Zijia Zheng: data curation; Ruisi Liu: investigation; Kejun Dong: methodology, writing – review & editing; Donghui Huang: methodology, writing – review & editing; Xianjin Xiao: methodology, supervision, writing – review & editing, funding acquisition, resources.

## Conflicts of interest

The authors declare no conflict of interests.

## Supplementary Material

RA-OLF-D6RA01614F-s001

## Data Availability

Data sharing is not applicable to this article as no new data were created or analyzed in this study. Supplementary information (SI) is available. See DOI: https://doi.org/10.1039/d6ra01614f.
