# Supplementary material for: Nanotechnology in reproductive medicine: from gamete engineering to precision therapeutics
Source: RSC Adv. 2026 Jul 8;16(35):34319–38. doi: 10.1039/d6ra01614f (PMC13343535; doi:10.1039/d6ra01614f)
Supplement: RA-016-D6RA01614F-s001 [file RA-016-D6RA01614F-s001.pdf]

Supplementary Information (SI) for RSC Advances.
This journal is © The Royal Society of Chemistry 2026

Supporting Information for

Nanotechnology in Reproductive Medicine: From Gamete Engineering to Precision Therapeutics

Xu Wen[a,1], Zhiyan Wang[b,1], Jiahui Lin[c,d,1], Longjie Li[e], Haiyun Wang[a], Pei Liu[a], Hao Hu[a], Chao He[a], Zijia Zheng[a], Ruisi Liu[a], Kejun Dong[f]*, Donghui Huang[a]*, Xianjin Xiao[a]*

a Institute of Reproductive Health, Tongji Medical College, Huazhong University of Science and Technology, Wuhan 430030, China

b The Second Clinical School, Tongji Hospital, Tongji Medical College, Huazhong University of Science and Technology, Wuhan 430030, China

c Reproductive Medical Center, Zhongnan Hospital of Wuhan University, Wuhan 430071, China;

d Hubei Provincial Key Laboratory of Developmentally Originated Diseases, Wuhan 430071, China.

e School of Life Science and Technology, Wuhan Polytechnic University, 430023, Wuhan, China

f Department of Obstetrics and Gynecology, Union Hospital, Tongji Medical College, Huazhong University of Science and Technology, Wuhan, 430022, China

1 Xu Wen, Zhiyan Wang, and Jiahui Lin are the authors who contribute equally.

Corresponding Authors:

Xianjin Xiao

Institute of Reproductive Health, Tongji Medical College, Huazhong University of Science and Technology, Wuhan 430030, China

xiaoxianjin@hust.edu.cn

Donghui Huang

Institute of Reproductive Health, Tongji Medical College, Huazhong University of Science and Technology, Wuhan 430030, China
jhsyyjs@126.com
Kejun Dong
Department of Obstetrics and Gynecology, Union Hospital, Tongji Medical College, Huazhong University of Science and Technology, Wuhan, 430022, China
540151069@qq.com

Table S1. Comparative summary of representative nanoplatforms in reproductive medicine

No.	Nanoplatform	Material type and size/composition	Main mechanism	Reproductive indication/application	Model system	Key outcomes	Main limitations	Translational stage
1	CeO_2NPs, SeNPs, ZnONPs	Inorganic metallic or metal oxide nanoparticles; composition-dependent antioxidant activity	ROS scavenging; antioxidant enzyme mimicry (catalase/SOD); reduced lipid peroxidation	Sperm motility improvement; sperm protection; cryopreservation support	Animal sperm models; heat stress and cryopreservation models	Improved motility, mitochondrial function, membrane integrity, and antioxidant enzyme activity	Dose-dependent toxicity; potential germ-cell damage; limited human data	Preclinical (animal and ex vivo)
2	Magnetic nanoparticles and sperm micromotors	Iron oxide-based magnetic nanoparticles or hybrid micromotor systems	Magnetic guidance; remote control of sperm movement; cargo delivery potential	Sperm function restoration; assisted sperm transport	In vitro sperm manipulation models	Directional sperm movement; externally controlled propulsion	Device complexity; uncertain reproductive safety; limited clinical applicability	Proof-of-concept / preclinical
3	Exosomes for sperm protection	Natural extracellular vesicles (phospholipid bilayer, ~40–150 nm)	Delivery of proteins, lipids, and miRNAs; antioxidant protection; membrane fusion	Sperm motility enhancement; cryodamage repair; varicocele-related dysfunction	Human and animal sperm models; varicocele rat model	Reduced ROS and apoptosis; improved sperm motility, vitality, and morphology	Difficult isolation; heterogeneity; limited large-scale production	Preclinical (biologically promising)
4	Nano-vitamins	Vitamin E nanoemulsions; vitamin C nanoparticles	Antioxidant protection against ROS-induced sperm damage	Sperm quality improvement; cryoprotection	Animal-assisted reproductive models	Reduced ROS; improved sperm motility, survival, and count	Limited clinical evidence; insufficient safety data	Preclinical (animal-based)
5	Lipid nanoparticles and cationic nanocarriers	Lipid nanoparticles; cellulose-based cationic complexes	mRNA/protein delivery; restoration of spermatogenesis-related pathways	Genetic male infertility (protein/gene defects)	Dmc1- and Pin1-deficient mouse models	Partial restoration of spermatogenesis and sperm function	Germline safety concerns; delivery specificity; long-term effects unclear	Preclinical (gene delivery stage)
6	Magnetic-activated cell sorting (MACS)	Annexin V-conjugated magnetic microspheres	Removal of apoptotic sperm via phosphatidylserine targeting	Sperm selection before ART/ICSI	Human semen samples; clinical ART studies	Reduced DNA fragmentation; improved chromatin maturity	Incomplete removal; possible loss of motile sperm; uncertain clinical benefit	Early clinical/laboratory use
7	Microfluidic sperm selection platforms	Microfluidic chips and sperm sorting systems	Selection based on motility, hydrodynamics, pH, and DNA integrity	High-quality sperm selection for ART	Human semen samples; in vitro sorting systems	Reduced ROS damage; improved motility and DNA integrity; enhanced embryo outcomes	Lack of standardization; cost; integration into clinical workflow	Early clinical translation
8	Follicular fluid-derived EVs and reproductive	Extracellular vesicles carrying miRNAs, proteins, and lipids	Intercellular communication; regulation of cumulus	Oocyte maturation; follicular microenvironment regulation	Mouse COCs; bovine EVs; reproductive tract models	Enhanced cumulus cell migration; improved oocyte maturation signaling	Isolation challenges; cargo heterogeneity; limited scalability	Preclinical

No.	Nanoplatform	Material type and size/composition	Main mechanism	Reproductive indication/application	Model system	Key outcomes	Main limitations	Translational stage
	exosomes		expansion and oocyte maturation					
9	Chitosan-based and lipid-based nanoparticles	Chitosan-TPP NPs; lipid carriers; peptide nanoparticles	Controlled release; gene/protein delivery; antioxidant effects	Oocyte maturation; IVM optimization; oxidative stress reduction	Animal IVF and oocyte models	Increased hormone levels; reduced oxidative stress; improved oocyte quality	Safety concerns; release control; reproducibility issues	Preclinical
10	Metallic nanoparticles for oocyte protection	Fe_3O_4, CeO_2, AuNPs, ZnO nanoparticles	ROS regulation; anti-apoptotic effects; mitochondrial protection	Oocyte quality improvement; PCOS-related ovarian dysfunction	PCOS and diabetic animal models; oocyte culture systems	Improved follicle growth; reduced apoptosis; enhanced fertilization potential	Potential ovarian toxicity; accumulation effects	Preclinical
11	3D hydrogel and nanofiber scaffolds	Alginate, fibrin, hyaluronic acid hydrogels; electrospun nanofibers	ECM mimicry; structural support; angiogenesis promotion	Ovarian repair; follicle culture; POI/POF treatment	In vitro follicle culture; animal ovarian models	Improved follicle survival; enhanced vascularization; delayed ovarian aging	Stability issues; degradation control; biosafety concerns	Preclinical
12	Nano-assisted cryopreservation systems	Graphene oxide, MOFs, quantum dots, metallic nanoparticles	Ice inhibition; magnetic/photothermal rewarming; cryoprotection	Gamete, embryo, and ovarian tissue preservation	Embryo and ovarian tissue models	Improved survival and developmental potential; reduced cryoinjury	Cytotoxicity at high doses; rewarming uniformity challenges	Preclinical
13	Engineered EVs for POI	Bioengineered extracellular vesicles (PD-L1, Gal-9)	Immune modulation; restoration of ovarian homeostasis	Premature ovarian insufficiency	POI animal models	Restored AMH levels; improved ovarian function	Production and standardization challenges; safety concerns	Preclinical
14	GO/PLLA nanofiber scaffold	Graphene oxide/polylactic acid composite scaffold	Structural support; ovarian tissue transplantation enhancement	Premature ovarian failure; fertility preservation	Mouse POF model	Increased AMH and estradiol; improved follicle count	Surgical dependence; long-term safety unclear	Preclinical
15	Senolytic nanoparticle systems	Nanocarriers for dasatinib and quercetin	Anti-senescence; ROS reduction; improved drug delivery	Chemotherapy-induced POF; ovarian aging	Cyclophosphamide-induced POF model	Improved oocyte quality; reduced DNA damage and apoptosis	Off-target effects; reproductive safety concerns	Preclinical
16	Vaginal/topical nanomedicine for PCOS	Nanogels; mucus-penetrating nanoparticles; lipid carriers	Local drug delivery; controlled release; improved mucosal penetration	PCOS; ovulatory dysfunction; metabolic disorders	PCOS animal models	Reduced ovarian weight; improved drug delivery; alleviated symptoms	Limited translation to human PCOS; local irritation risk	Preclinical
17	Bioactive nanoparticles for PCOS	Metal-polymer hybrids; chitosan-based nanoparticles	Anti-inflammatory; antioxidant; enhanced cellular uptake	PCOS-related inflammation and oxidative stress	Cell models and PCOS animal models	Reduced inflammatory cytokines; improved biochemical parameters	Potential toxicity; lack of standardization	Preclinical
18	NIR-II nanoprobes	Rare-earth-based	High-resolution	Diagnosis of tubal	Preclinical imaging	Enhanced imaging contrast;	Clinical safety and	Preclinical

No.	Nanoplatform	Material type and size/composition	Main mechanism	Reproductive indication/application	Model system	Key outcomes	Main limitations	Translational stage
	for tubal disease	fluorescent nanoparticles	fluorescence imaging	obstruction and hydrosalpinx	models	improved visualization	clearance unclear	
19	Hydrogel systems for tubal repair	Stem cell-loaded or ECM-derived hydrogels	Tissue regeneration; biomimetic microenvironment	Tubal repair; embryo culture	Tubal injury and embryo models	Improved tissue repair; enhanced embryo environment	Early-stage validation; mechanical properties unresolved	Preclinical
20	Imaging nanoplatforms for endometriosis	Fluorescent, MRI, and targeted nanoparticles	Targeted imaging; photoacoustic detection	Endometriosis diagnosis and intraoperative guidance	Animal endometriosis models	Improved lesion detection and retention	Potential toxicity; limited human validation	Preclinical
21	Therapeutic nanoplatforms for endometriosis	Multifunctional nanoparticles; RNAi systems	ROS modulation; anti-angiogenesis; gene silencing	Endometriosis treatment	Animal models; uterine models	Reduced lesion size; decreased inflammation and angiogenesis	Limited clinical evidence; safety concerns	Preclinical
